# Distribution and abundance of key vectors of Rift Valley fever and other arboviruses in two ecologically distinct counties in Kenya

**DOI:** 10.1371/journal.pntd.0005341

**Published:** 2017-02-17

**Authors:** Rosemary Sang, Samwel Arum, Edith Chepkorir, Gladys Mosomtai, Caroline Tigoi, Faith Sigei, Olivia Wesula Lwande, Tobias Landmann, Hippolyte Affognon, Clas Ahlm, Magnus Evander

**Affiliations:** 1 International Centre of Insect Physiology and Ecology, Nairobi, Kenya; 2 Center for Virus Research, Kenya Medical Research Institute, Nairobi, Kenya; 3 Department of Clinical Microbiology, Virology, Umeå University, Umeå, Sweden; 4 International Crops Research Institute for the Semi-Arid Tropics (ICRISAT), Bamako, Mali; 5 Department of Clinical Microbiology, Infectious Diseases, Umeå University, Umeå, Sweden; School of Veterinary Medicine University of California Davis, UNITED STATES

## Abstract

**Background:**

Rift Valley fever (RVF) is a mosquito-borne viral zoonosis of ruminants and humans that causes outbreaks in Africa and the Arabian Peninsula with significant public health and economic consequences. Humans become infected through mosquito bites and contact with infected livestock. The virus is maintained between outbreaks through vertically infected eggs of the primary vectors of *Aedes* species which emerge following rains with extensive flooding. Infected female mosquitoes initiate transmission among nearby animals, which amplifies virus, thereby infecting more mosquitoes and moving the virus beyond the initial point of emergence. With each successive outbreak, RVF has been found to expand its geographic distribution to new areas, possibly driven by available vectors. The aim of the present study was to determine if RVF virus (RVFV) transmission risk in two different ecological zones in Kenya could be assessed by looking at the species composition, abundance and distribution of key primary and secondary vector species and the level of virus activity.

**Methodology:**

Mosquitoes were trapped during short and long rainy seasons in 2014 and 2015 using CO_2_ baited CDC light traps in two counties which differ in RVF epidemic risk levels(high risk Tana-River and low risk Isiolo),cryo-preserved in liquid nitrogen, transported to the laboratory, and identified to species. Mosquito pools were analyzed for virus infection using cell culture screening and molecular analysis.

**Findings:**

Over 69,000 mosquitoes were sampled and identified as 40 different species belonging to 6 genera (*Aedes*, *Anopheles*, *Mansonia*, *Culex*, *Aedeomyia*, *Coquillettidia*). The presence and abundance of *Aedes mcintoshi* and *Aedes ochraceus*, the primary mosquito vectors associated with RVFV transmission in outbreaks, varied significantly between Tana-River and Isiolo. *Ae*. *mcintoshi* was abundant in Tana-River and Isiolo but notably, *Aedes ochraceus* found in relatively high numbers in Tana-River (n = 1,290), was totally absent in all Isiolo sites. Fourteen virus isolates including Sindbis, Bunyamwera, and West Nile fever viruses were isolated mostly from *Ae*. *mcintoshi* sampled in Tana-River. RVFV was not detected in any of the mosquitoes.

**Conclusion:**

This study presents the geographic distribution and abundance of arbovirus vectors in two Kenyan counties, which may assist with risk assessment for mosquito borne diseases.

## Author summary

Rift Valley fever (RVF) is a mosquito-borne disease caused by the Rift Valley fever virus (RVFV) transmitted by diverse species of mosquitoes broadly classified into primary vectors and secondary vectors. Primary vectors consist of floodwater *Aedes* (e.g *Ae*. *mcintoshi*, *Ae*. *ochraceus*, *Ae*. *sudanensis*, *Ae*. *dentatus* etc), known to maintain the virus in their drought resistant eggs which are deposited on wet soils on low lying depressions on land, remaining viable in dry soil for variable number of years during dry periods. Following heavy persistent rains with flooding, such eggs hatch with a proportion already infected. Emerging infected adult female mosquitoes initiate transmission to nearby animals which serve as amplifiers, infecting more mosquitoes resulting in outbreaks. Another group of mosquito species, the secondary vectors, mainly from the *Culex* (*Culex pipiens* and *Culex poicilipes*), and other potential vectors including, *Culex univittatus*, *Anopheles* and *Mansonia* species may take over such breeding sites, breed in abundance and incidentally propagate RVFV transmission. Outbreaks of RFV occur at varying intensities among livestock in different counties in Kenya, and counties are classified into high, medium and low risk zones. We assessed the species composition, distribution and abundance of primary and secondary vectors in two counties; Isiolo (medium risk) and Tana-River (high risk). Striking difference in composition of primary vector species between Isiolo and Tana-River was observed suggesting that vector species composition in different regions could further be applied to assess risk of RVF outbreaks and intensity. We propose further evaluation of vector species surveillance as an additional risk assessment tool for RVFV and other mosquito borne viruses.

## Introduction

Rift Valley fever virus (RVFV), of the genus *Phlebovirus*, family *Bunyaviridae* is a mosquito-borne virus present in Africa and the Arabian Peninsula [1]. It causes disease of varying severity including hemorrhagic fever, encephalitis and mortalities in humans and abortions and death among ruminants. RVF outbreaks occur in many parts of Africa every 5 to 15 years during periods of heavy and persistent rainfall that often leads to flooding.

Animals are mainly infected through bites of infected mosquitoes, while humans are typically exposed when they come in direct contact with infected bodily fluids or tissues of infected animals. Transmission to humans via mosquito bites is speculated to cause milder disease or asymptomatic infections [2,3].

Since being first identified in the 1930s, recurring RVF outbreaks have led to high morbidity and mortality in humans and livestock as well as significant economic loss in affected regions/countries [3]. The outbreaks that affected eastern Africa in 1997/98 and 2006/2007 were most widespread in Kenya, Tanzania, Somalia, Djibouti, Sudan and South Sudan and although the full impact of the outbreaks in terms of public health and economic loss for the entire region may not have been fully assessed, it is documented that Kenya suffered losses to the extent of US $ 32 million due to losses of animal herds, vaccination costs and trade bans/value chain ramifications[4]. During the 2006/2007 outbreak there were more than 150 reported human deaths due to RVFV and over 700 human cases, and there was strain on the already overstretched public health resources and facilities in the North-Eastern regions of Kenya [5].

Mosquitoes collectively referred to as floodwater *Aedes* have been classified as the primary vectors of RVF, maintaining the virus through transovarially infected drought, resistant eggs that survive in dry soils on low lying depressions on land over inter-epidemic periods that could be as long as 5 to 15 years. However, it is also suspected that inter-epidemic period may last for more than 15 years in some regions [6,7]. Flooding due to heavy persistent rainfall results in mass emergence of flood water *Aedes* mosquitoes. Vertically infected (infected eggs) that emerge initiate virus transmission to nearby animals, which could lead to an outbreak depending on continued precipitation and flooding of vector breeding habitats and elevated abundance of vectors [6]. Other mosquitoes in the *Culex*, (generally referred to as secondary vectors) and other potential secondary vectors such as *Anopheles and Mansonia* genera succeed the primary vector species taking over the flooded grounds to further support virus transmission in the later part of the outbreak period [8]. During investigations of the 2006/2007 RVF outbreak in Kenya, 10 mosquito species principally *Ae*. *mcintoshi*, *Ae*. *ochraceus* (primary vectors), and a range of other secondary vector species, sampled in ecologically diverse affected regions (including Garissa and Tana-River) were found positive for RVFV [9].

Eleven national epizootics of RVF have occurred in Kenya between 1951and 2007; 8 (12%) districts being affected in 1951, 22 (32%) in 1961–64 (including Garissa, Tana River and Isiolo) and 48% (33/69) in the 2006/2007 outbreak period [10]. Thus the geographic expansion of RVF is increasing with each successive outbreak and, apart from environmental drivers (rainfall and temperature) and the density and movement of livestock, the presence of competent vector species is very important for virus transmission to occur and to be established in any new area [6, 11]. Transmission via infected mosquitoes remains crucial for the dissemination of RVFV between herds or flocks over short and long distances allowing for the emergence and dissemination of the disease throughout a region or a country preceded by the movement of infected animals [11].

The sensitivity and specificity of disease risk assessment and forecasting may be improved by characterizing more small scale and explicit factors that are associated with varying disease occurrences in certain regions within a country. To generate data that would improve assessment of disease risk and regional vulnerability, we investigated the composition and distribution of known vectors of RVFV in two counties, namely Tana-River and Isiolo, known to have different ecologic settings and different levels of disease activity. In 2006/2007, Tana-River suffered a significantly higher impact RVF with 7 deaths out of 16 reported human cases compared to Isiolo with 0 deaths out of 7 probable cases and in addition, Tana-River and Isiolo have been classified as being at high and medium risk of RVF respectively, based on livestock infection data [5, 12]. We also investigated presence of circulating arboviruses in the mosquito population.

## Methods

### Study sites

This study was implemented in the Tana-River and Isiolo counties of Kenya, selected based on the differential impact of the RVF outbreak in 2006/2007. Tana-River was more affected with16 human cases than Isiolo that only reported 7 probable cases [5, 12].

Tana-River County; borders Garissa County to the west, covers 38,437 km^2^ and has a coastal strip of 35 km. The county is composed of three sub-counties; Bura, Galole and Garsen and has a population of 240,075, according to the 2009 census distributed in 47,414 households. It is inhabited by a mixture of ethnic Orma and Somali communities that practice pastoral farming, with large herds of livestock, consisting mainly of cattle, sheep and goats. Riverine forest, woodland, grassland, bush lands, lakes, open river channels, sand dunes, mangroves and coastal waters are among the diverse ecologies broadly classified under the semi arid and semi humid ecological zones in Tana-River. The county is generally dry and prone to drought. Rainfall is erratic, with rainy seasons falling in March–May and October–December while mean annual rainfall amounts vary between400mm and 750mm. The mean annual temperature ranges between 30°C and 33°C. Tana-River has been classified as being at high risk for RVF outbreaks [12] and it suffered a significantly high impact with 7 deaths out of 16 reported probable cases during the 2006/2007 outbreak period [5] although these figures may be considered an underestimation as some cases may have been missed due to various reasons including poor access to health facilities and challenges of identifying cases.

Isiolo county; is an expansive county (25,336 km^2^) inhabited by diverse ethnic communities. Although the population is predominantly Cushite communities (Oromo-speaking Boran and Sakuye) there are Turkana, Samburu, Meru, Somali and other immigrant communities from other parts of the country. Borana form the largest proportion and except for the Meru, the rest of the communities practice pastoralism. Isiolo has three ecological zones; semi-arid, arid and the very arid. The semi-arid zone makes 5% of the county with an annual rainfall of between 400–650mm. The relatively high rainfall here is due to the influence of mount Kenya and Nyambene Hills in the neighbouring Meru County. However, 95% of the county falls in the arid to very arid zone. Isiolo suffered RVF outbreak at a smaller scale than Tana-River and in the most recent RVF risk classification for Kenya by counties, Isiolo was classified as being at medium risk for RVF outbreaks [5, 12]. In the scarce data available of RVF cases during outbreaks of 2006/2007, Isiolo documented no deaths out of 7 probable cases [5] although again there may have been significant under- reporting. For this study, sampling in all sites was performed following long and short rains to target periods of possible vector activity and RVF transmission.

### Sampling and identification of mosquito vectors

**Fig 1:**
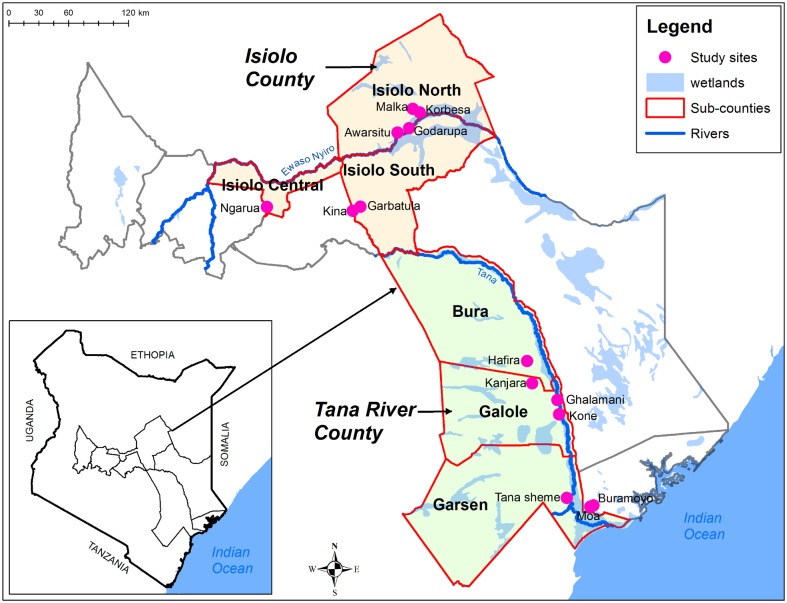
Map of vector sampling sites in Isiolo and Tana-River counties, Kenya.

Mosquitoes were trapped using CO_2_-baited CDC light traps (John W. Hock Company-Model 512) twice every year at the selected study sites in Tana-River and Isiolo areas (Fig 1) during the long rains (April–June) and short rains (November–December) between 2014 and 2015, respectively. There were a total of seven sampling sites in each area cutting through a transect of all the sub-counties and ecological zones (Fig 1).

These sites were selected along the major livestock movement routes used by nomadic herders in both regions and also represented the different ecozones in each of the two major sites. During each trapping period and in each site, ten traps were set at 1800 hrs and retrieved at 0600 hrs the following day for three consecutive sampling days in both seasons. Trapped mosquitoes were anesthetized using triethylamine (Sigma-Aldrich-471283) for ten minutes, separated from other insects, placed into 15 ml labeled tubes, and transported to the laboratory in liquid nitrogen where they were stored at -80°C and subsequently morphologically identified to species level using available taxonomic keys [13–16]. Mosquitoes were grouped in pools of up to 25 mosquitoes belonging to the same species, sex, collection date and trap and stored to be homogenized and analyzed for viruses.

### Arbovirus isolation, characterization and analysis by RT-PCR

Mosquito homogenates were prepared from identified mosquito pools for virus isolation and characterization following previously published standard procedure [17, 18]. Homogenates were transferred to a 1.5 ml cryovial and stored at -80°C ready for testing. Homogenates were screened for viruses by inoculation of 50 μl of each pool into a monolayer of Vero E6 cells (monkey kidney continuous cell line) grown in 24 well plates following previously published standard virus isolation procedure [17, 18].

Samples giving reproducible CPE were processed for molecular analysis to determine the identity of the virus isolate following previously published procedures and using available primer sets that flank conserved regions of African arbovirus species or families [17, 18]. The PCR cycling conditions varied for each specific virus. The specific reactions were conducted using cycling condition for specific primers for virus genus (alpha viruses, flaviviruses and orthobunya viruses) and virus type (RVFV, Bunyamwera, West Nile, Sindbis, Batai).

### Statistical analysis

The mosquito species diversity and density data were analyzed using R version 3.1.1 [19, 20]. The differences in the proportions of the total captures for mosquito species between the areas (Isiolo and Tana-River) were evaluated using generalized linear models (GLM). Quasi-poisson regression was used to test significant difference between the vector groups and individual species.

## Results

A total of 69,103 mosquitoes were sampled, identified, and stratified into 40 different species belonging to 6 genera including *Aedes*, *Anopheles*, *Mansonia*, *Culex*, *Aedeomyia*, and *Coquillettidia*. The vectors were categorized into three main groups; primary vectors for RVFV (*Ae*. *mcintoshi* and *Ae*. *ochraceus)*, secondary vectors (*Ae*. *sudanensis*, *An*. *squamosus*, *Ma*. *africana*, *Ma*. *uniformis*, *Cx*. *pipiens*, *Cx*. *univittatus* and *Cx*. *poicilipes*) and other mosquitoes including vectors of malaria (Table 1).

**Table 1:** Mosquito species collected across the study sites in Tana-River and Isiolo counties, Kenya.

Areas	Sites	Primary vectors		Secondary vectors							Other mosquitoes*											
		*Ae*. *mcintoshi*	*Ae*. *ochraceus*	*Cx*. *pipiens*	*Cx*. *poicilipes*	*Ma*. *africana*	*Ma*. *uniformis*	*Cx*. *univittatus*	*Ae*. *sudanensis*	*An*. *squamosus*	*Ae*. *cuminsi*	*Ae*. *tircholabis*	*Ae*. *hirsutus*	*Cx*. *vansomereni*	*An*. *gambiae*	*An*. *coustani*	*An*. *funestus*	*An*. *pharoensis*	*Cx*. *ethiopicus*	*Cx*. *tigripes*	*Cx*. *anulioris*	Total
**Tana-River**	Ghalamani	1389	31	967	58	858	3	243	42	5	34	502	0	558	3	8	4	0	0	3	9	4717
	Kone	1324	79	1107	15	39	14	68	49	16	0	504	0	1138	22	9	9	0	0	0	3	4396
	Hafira	780	66	347	14	24	4	69	71	0	0	179	0	1760	5	2	2	0	0	0	0	3323
	Kanjara	1188	1033	133	1	1	0	83	430	1	0	615	0	0	0	0	0	0	5	0	0	3490
**Tana-Rive** (Garsen subcounty)	Tana scheme	313	14	1224	11	115	2	35	37	206	0	22	0	851	34	6	68	46	7	3	2015	5009
(Garsen subcounty)	Buramoyo	339	14	734	3	58	0	19	16	3	0	31	0	123	1	1	12	52	7	5	2530	3948
(Garsen subcounty)	Moa	590	53	2370	10	80	0	25	64	23	0	11	0	1152	47	3	125	81	7	8	3133	7782
**Sub-total (n)**		**5923**	**1290**	**6882**	**112**	**1175**	**23**	**542**	**709**	**254**	**34**	**1864**	**0**	**5582**	**112**	**29**	**220**	**179**	**26**	**19**	**7690**	**32665**
Proportion of species/total (%)		18	3.9	21	<1	3.6	<1	1.7	2.2	<1	<1	5.7	0	17	<1	<1	<1	<1	<1	<1	24	
**Isiolo C**	Ngarua	8	0	3798	47	3	3	2880	0	7	0	8	9	62	521	103	15	21	182	3	175	7845
**Isiolo S**	Kina	943	0	360	0	6	2	694	5	3	0	411	1137	2	681		433	4	11	1	3	4714
	Garbatula	995	0	147	0	0	1	1876	3	1	0	426	42	2	139	2	6	8	3	7	2	4388
**Isiolo N**	Godarupa	1961	0	290	1	2	4	2001	5	6	68	951	33	289	721	1	5	14	1	0	30	6383
	Korbesa	793	0	73	0	1	1	408	0	3	68	464	12	63	867	0	7	2	1	0	0	2035
	Awarsitu	501	0	78	3	0	1	840	0	1	0	245	1	0	521	2	9	0	34	0	0	2236
	Malka	940	0	129	0	0	0	2070	4	1	0	621	0	15	628	0	1	8	0	0	4	4421
**Sub-total (n)**		**6141**	**0**	**4875**	**51**	**12**	**12**	**10769**	**17**	**22**	**136**	**3126**	**1234**	**433**	**4078**	**126**	**476**	**57**	**232**	**11**	**214**	**32022**
**Proportion of species/total (%)**		**20**	**0**	**16**	**<1**	**<1**	**<1**	**35**	**<1**	**<1**	**<1**	**10**	**3.8**	**1.4**	**13**	**<1**	**1.4**	**<1**	**<1**	**<1**	**<1**	
**Relative Proportions(Isiolo/Tana River)**		**1.04**	**0**	**0.70**	**0.45**	**0.01**	**0.52**	**19.80**	**0.02**	**0.08**	**4**	**1.67**	**-**	**0.07**	**36.41**	**4.34**	**2.16**	**0.32**	**8.92**	**0.58**	**0.03**	**0.94**

Isiolo C, Isiolo Central; Isiolo S, Isiolo South; Isiolo N, Isiolo north *Ae*., *Aedes*; *Cx*., *Culex; Ma*., *Mansonia; An*., *Anopheles*.

*Only single mosquitoes of the *Aedeomyia* and *Coquillettidia* genera were collected and are not shown in the table

Of the total mosquitoes collected during the entire sampling period from the two regions, the RVFV primary vectors constituted 20.60% (n = 13,354) while the RVFV secondary vectors constituted 39% (n = 25,455). It was also notable that other mosquito species including secondary vectors were more abundant in Tana River (n = 15,755), than in Isiolo (n = 10, 123). These species comprised *Cx*. *poicilipes* and *Cx*. *pipiens* secondary vectors of RVF, and *Cx*. *univittatus* which are known vectors of WNV and SNV.

Overall comparison of the vector groups showed that there was no significant difference in distribution of the primary RVFV vectors *Ae*. *mcintoshi* and *Ae*. *ochraceus* (F_1, 12_ = 0.200, P = 0.662) and secondary vectors of RVF including *Cx*. *poicilipes* and *Cx*. *pipiens*, and potential vector, *Cx*. *univittatus* (F_1, 12_ = 1.213, P = 0.292) across Isiolo and the Tana-River regions. *Aedes mcintoshi*, one of the two most important floodwater *Aedes* species associated with RVFV transmission in the last outbreak of 2006/2007,was abundant in both Tana-River and Isiolo but much more so in Tana-River. In contrast, *Ae*. *ochraceus*, the other important species that was found to have played a key role in the last outbreak, was only found to be present in the Tana-River sites (n = 1,291), while totally absent in all the samples collected from all sites in Isiolo. There was no significant difference in the number of *Ae*.*mcintoshi* sampled trapped from the two areas (F_1, 12_ = 0.012, P = 0.913). The different captures of the mosquitoes across the sampling sites is presented in Table 1. There was a significant difference in captures of the secondary vectors of RVFV; *Ae*. *sudanensis*, *Ma*. *africana* and *Cx*. *univittatus* across the different sites. Another floodwater *Aedes* species, *Aedes tricholabis* which has never been associated with RVFV transmission/maintenance was sampled in high numbers in both Tana-River and Isiolo. Significantly higher captures of *Ae*. *sudanensis* (F_1,12_ = 7.927, P = 0.015) and *Ma*. *africana* (F_1,12_ = 5.370, P = 0.038) were obtained in Tana-River while capture of *Cx*. *univittatus* was significantly higher in Isiolo (F_1,12_ = 5.220, P<0.001).

Among the Anophelines, *Anopheles squamosous* previously reported to be infected with RVFV and was potentially associated with the RVF outbreak in Kenya in 2006/2007) [9] was more abundant at one site in Tana-River while in Isiolo it was only occasionally found. The important malaria vectors *An*. *gambiae* and *An*. *funestus* were most abundant in Isiolo.

### Virus isolations and identity

A total of 4,636 mosquito pools representing collections sampled from the different sites and sampling dates were screened for viruses, and 14 virus isolates were obtained from 14 mosquito pools. By conventional RT-PCR the isolates were shown to include seven SINV: five from *Ae*. *mcintoshi* (Ghalamani, Tana-River); one from *Cx*. *pipiens* (Ghalamani, Tana-River); one from a *Culex* species (Kone, Tana-River); one WNV isolated from *Cx*. *vansomereni* (Kone, Tana-Riverone *Orthobunyavirus* isolated from *Ae*. *mcintoshi* (Ghalamani, Tana-River).

Two isolates from *Ae*.*mcintoshi* (Ghalamani, Tana-River), one from *Ae*. *furfurea*(Kone, Tana-River), and one from *Ae*. *tricholabis* (Ghalamani, Tana-River) remained unidentifiable despite attempts using available arbovirus primers. An *Orthobunyavirus* virus isolate was obtained from *Cx*. *vansomereni* from Ngarua, the only viral isolate from all Isiolo mosquito samples. Seven isolates (five SINV, one WNV, one *Orthobunyavirus*) were confirmed by PCR with respective primers (S1 File).

## Discussion

The mosquito species sampled in the study sites revealed differences in species composition and abundance that could influence the differential epidemic impact of RVF in the two counties of Isiolo and Tana-River. The observed difference in the distribution of vectors between the two regions could be attributed to the ecology and habitats of the regions. Entomologic investigations performed during the RVF outbreak 2006/2007 in Kenya, together with previous field and laboratory studies incriminated a number of mosquito species as primary and secondary vectors of RVF through virus detection in wild caught specimens including *Ae*. *mcintoshi*, *Ae*. *ochraceus*, *Ae*. *dentatus* to name a few primary vectors and RVFV was also detected in wild caught *Cx*. *pipiens*, and *Cx*. *poicilipes*, secondary vectors of RVF virus including potential secondary vectors; *Cx*. *univittatus* and *Cx*. *vansomereni*, [6, 9, 21].

During the 2006/2007 RVFV outbreak the other important primary vector identified was *Ae*. *ochraceus* [9] which in the present study was found in significantly lower numbers compared to *Ae*. *mcintoshi* in Tana-River. However, the most striking observation was the total absence of *Ae*. *ochraceus* in all the Isiolo sites sampled. It is possible that this species, which was abundantly sampled in Garissa and Tana-River during the RVF outbreak in 2006/2007 and found commonly infected with RVF virus is yet to expand its geographic spread to Isiolo and possibly to other counties in Kenya. Indeed, recent population genetic studies conducted on representative samples of *Ae*. *ochraceus* sampled from various sites in north-eastern Kenya and West Africa affected by RVF outbreaks revealed that *Ae*. *ochraceus* constituted a recently introduced species to Kenya [22].

Our findings further corroborate this observation suggesting that the species is yet to colonise parts of Kenya and possibly the Eastern African region fully. Most RVF epidemics have been linked to *Ae*. *mcintoshi* as the key primary vector. Experimental studies have been conducted on *Ae*. *mcintoshi* whose significant role in RVF transmission have been suggested through virus isolation from samples collected from the wild. However, it was found to be an inefficient vector exhibiting a major salivary gland barrier with only 14% of the mosquitoes which developed disseminated infection transmitted virus by bite [23]. The importance of this mosquito as key RVF vectors could potentially be attributed to its abundance and feeding patterns in RVF prone regions. Apart from the multiple detections of RVFV in wild caught specimens during outbreaks [9, 24], there are no studies done to determine the efficiency of *Ae*. *ochraceus* in transmitting RVFV.

We can speculate that the explosive outbreaks of RVF experienced in Tana-River and Garissa could be attributed to the possible role of *Ae*. *ochraceus*, supporting *Ae*. *mcintoshi*, since both species were found to be infected with the virus during the outbreak in 2006/2007[9]. Exploring the distribution and vectorial capacity of *Ae*. *ochraceus* in other high and medium risk zones could shed more light on this.

The densities of other mosquito species which also transmit RVFV and other arboviruses, including *Cx*. *pipiens* and *Cx*. *univittatus*, were however, found in significantly greater numbers in Isiolo compared to Tana-River. These are among the *Culex* species from which the RVFV was isolated during previous outbreaks [9, 25]. The efficiency of *Cx*. *univittatus* in transmitting RVFV has not been explored fully but experimental studies on a member of the complex, *Cx*. *perexiguus* has shown this to be an efficient vector of RVFV [26]. Although the species feed more readily on birds, it also feed opportunistically on humans, and where RVF virus transmission has been initiated by the primary vectors, secondary vectors such as *Cx*. *poicilipes* and *Cx*. *pipiens*, other *Culex* species including *Cx*. *vansomereni* and *Cx*. *univittatus*,can potentially play a role in transmitting the virus among humans and even to animals [27]. Thus, RVFV could spread widely in human populations in Isiolo, following initial transmission among livestock by floodwater *Aedes*, and would require vector control efforts targeting *Cx*. *pipiens* species to be put in place in order to break human to human transmission and reduce public health impact. Other potential secondary vectors like the *Mansonia* and *Anopheles* species were more predominant in Tana-River than in Isiolo including *Ma*. *uniformis*, *Ma*.*africanus* and *An*. *squamosus*, all of which were found infected with RVFV during the 2006/2007 RVF outbreak in Kenya [9].

This study has demonstrated clear differences in vector species composition and abundance in two counties of diverse ecologies and epidemic risks and we suggest that this approach could be used in determining risk levels for transmission and outbreaks of RVF to augment the other currently used ecological risk factors. Efforts to isolate and detect RVFV circulation among the sampled vectors both in Isiolo and Tana-River sites did not yield any isolate. This is in spite of low level circulation of the RVFV noted through monitoring of livestock migrating through these regions [28]. Previous efforts to isolate RVFV from vectors in the inter-epidemic period have not been successful [29] and we suggested that outside of outbreaks, RVFV circulates in vectors at levels that are below detection in terms of minimum infection rates (infection rates per 1000 mosquitoes tested). During the 2006/2007 outbreak [9], minimum infection rates in mosquito species sampled ranged between 0.8 and 2.5, during which time RVFV was detected in 51 out of 1,038 mosquito pools of diverse species in Garissa county, thus during inter-epidemic period, RVFV isolation becomes very unlikely, requiring analysis of huge numbers of mosquitoes to be able to detect a single infected mosquito. However, other arboviruses were isolated from three virus genera, *Alphavirus*, *Flavivirus* and *Orthobunyavirus*. This study provides important information about the distribution of key vectors of RVF in Tana-River and Isiolo counties. Diversity and distribution of the vectors in two study sites could be one of the factors which could contribute differential patterns of RVF occurrence in the two regions.

These findings support previous studies on RVFV and risk factors associated with RVF outbreaks in Kenya [2, 6, 30]. Most of the viruses isolated were obtained from mosquitoes sampled in Tana-River and mostly from *Ae*. *mcintoshi* even though they were not the most abundant species in the collection. It is not known why most virus isolates were found in *Ae*. *mcintoshi* species but we speculate that due to their feeding preference, they may be infected while feeding on animals/birds which may serve as reservoirs and which mainly converge in Tana-River probably due to availability of fodder, water and pasture. Tana also serves as a major stopover and roosting site for local and migratory birds using the East African flyway [31]. Only one isolate was obtained from a single mosquito pool in Isiolo compared to Tana-River where 13 virus isolates were obtained. This could be attributed to the ecological differences between the regions as well as the diversity of potential vector species. Previous studies have also reported low numbers of arbovirus cases in Isiolo relative to Tana-River [5, 12]. In previous arbovirus surveys conducted in neighboring Garissa county, WNV, Semliki Forest virus and Ndumu virus, traditionally known to be mosquito-borne, were detected in ticks taken off cattle and other wild animals [32] suggesting that although these viruses have traditional reservoirs and vectors which have been documented, they may infect other animal species from which mosquitoes such as *Ae*. *mcintoshi*(known to feed preferentially on livestock) could acquire the virus making the network of arbovirus transmission even more complex and unconventional.*Culex* species from which WNV was isolated feed on diverse species including birds which are known reservoirs of SINV and WNV.

## Conclusion

This study demonstrated a marked difference in species composition, abundance and distribution of primary and secondary mosquito vector sof RVFV in two counties of Kenya, classified as being at high and medium risk for RVF outbreaks, respectively. Some of the vectors are known to be involved in RVFV maintenance and transmission suggesting that presence/absence of key RVFV vector species or combination of species could define disease risk, distribution and expansion. The need for RVFV vector competence evaluation for key species is needed to improve the risk evaluation further.

## Supporting information

S1 File Sequences of primers used for virus characterization. (DOCX)

## References

[1] Davies F. Observations on the epidemiology of Rift Valley fever in Kenya. Journal of Hygien (Lond). 1975;75(2):219–30. 10.1017/s0022172400047252 PMC2130298 1058243

[2] Hoogstraal H, Meegan JM, Khalil GM, Adham FK.The Rift Valley fever epizootic in Egypt 1977–1978 Ecological and entomological studies.Transactions of the Royal Society of tropical Medicine and Hygiene. 1979;73(6):624–9. 44038 10.1016/0035-9203(79)90005-1

[3] Pepin M, Bouloy M, Bird BH, Kemp A, Paweska J. Rift Valley fever virus (Bunyaviridae: Phlebovirus): an update on pathogenesis, molecular epidemiology, vectors, diagnostics and prevention. Veterinary Research. 2010;41(6):61 10.1051/vetres/2010033 21188836 PMC2896810

[4] Rich KM, Wanyoike F. An assessment of the regional and national socio-economic impacts of the 2007 Rift Valley fever outbreak in Kenya. The American Journal of Tropical Medicine and Hygiene. 2010;83(2 Suppl):52–7. 10.4269/ajtmh.2010.09-0291 20682906 PMC2913501

[5] Nguku PM, Sharif S, Mutonga D, Amwayi S, Omolo J, Mohammed O, et al An investigation of a major outbreak of Rift Valley fever in Kenya: 2006–2007. The American Journal of Tropical Medicine and Hygiene. 2010;83(2 Suppl):05–13. 10.4269/ajtmh.2010.09-0288 PMC2913496 20682900

[6] Linthicum K, Davies F, Kairo A, Bailey C. Rift Valley fever virus (family Bunyaviridae, genus Phlebovirus). Isolations from Diptera collected during an inter-epizootic period in Kenya. Journal of Hygiene. 1985;95(01):197–209. 2862206 10.1017/s0022172400062434 PMC2129511

[7] Manore C, Beechler B. Inter-Epidemic and Between-Season Persistence of Rift Valley Fever: Vertical Transmission or Cryptic Cycling? Transboundary and Emerging Diseases. 2013;62(1):13–23. 10.1111/tbed.12082 23551913 PMC5113711

[8] Davies F, Linthicum K, James A. Rainfall and epizootic Rift Valley fever. Bulletin of the World Health Organization. 1985;63(5):941 3879206 PMC2536443

[9] Sang R, Kioko E, Lutomiah J, Warigia M, Ochieng C, O'Guinn M, et al Rift Valley fever virus epidemic in Kenya, 2006/2007: the entomologic investigations. The American Journal of Tropical Medicine and Hygiene. 2010;83(2 Suppl):28–37. 10.4269/ajtmh.2010.09-0319 20682903 PMC2913497

[10] Murithi R, Munyua P, Ithondeka P, Macharia J, Hightower A, Luman E, et al Rift Valley fever in Kenya: history of epizootics and identification of vulnerable districts. Epidemiology and Infection. 2011;139(03):372–80. 20478084 10.1017/S0950268810001020

[11] Chevalier V, Mondet B, Diaite A, Lancelot R, Fall A, Ponçon N. Exposure of sheep to mosquito bites: possible consequences for the transmission risk of Rift Valley Fever in Senegal. Medical and Veterinary Entomology. 2004;18(3):247–55. 10.1111/j.0269-283X.2004.00511.x 15347392

[12] Munyua PM, Murithi RM, Ithondeka P, Hightower A, Thumbi SM, Anyangu SA, et al Predictive Factors and Risk Mapping for Rift Valley Fever Epidemics in Kenya. PloS One. 2016;11(1):e0144570 10.1371/journal.pone.0144570 26808021 PMC4726791

[13] Edwards FW. Mosquitoes of the Ethiopian Region. III.-Culicine adults and pupae. Mosquitoes of the Ethiopian Region III-Culicine Adults and Pupae. 1941.

[14] Gillies MT, De Meillon B. The anophelinae of Africa south of the Sahara (Ethiopian zoogeographical region). The Anophelinae of Africa south of the Sahara (Ethiopian Zoogeographical Region). 1968.

[15] Harbach RE. The mosquitoes of the subgenus Culex in southwestern Asia and Egypt (Diptera: Culicidae). Contributions of the American Entomological Institute. 1988;24(1).

[16] Jupp P. Mosquitoes of Southern Africa. Hartebeespoort. South Africa): Ekogilde Publishers; 1996.

[17] O’guinn ML, Lee JS, Kondig JP, Fernandez R, Carbajal F. Field detection of eastern equine encephalitis virus in the Amazon Basin region of Peru using reverse transcription-polymerase chain reaction adapted for field identification of arthropod-borne pathogens. The American Journal of Tropical Medicine and Hygiene. 2004;70(2):164–71. 14993628

[18] Ochieng C, Lutomiah J, Makio A, Koka H, Chepkorir E, Yalwala S, et al Mosquito-borne arbovirus surveillance at selected sites in diverse ecological zones of Kenya; 2007–2012. Virology Journal. 2013;10(1):1. 23663381 10.1186/1743-422X-10-140 PMC3669043

[19] Oksanen J, Blanchet F, Kindt R, Legendre P, Minchin P, O’Hara R, et al. vegan: Community Ecology Package. R package version 2.2–1. 2015.

[20] Kembel SW, Cowan PD, Helmus MR, Cornwell WK, Morlon H, Ackerly DD, et al Picante: R tools for integrating phylogenies and ecology. Bioinformatics. 2010;26(11):1463–4. 10.1093/bioinformatics/btq166 20395285

[21] Davies F H RB. Possible vector of Rift Valley fever in Kenya. Trans of The Royal Society of Tropical Medicine and Hygiene; 1980 p. 815–25. 10.1016/0035-9203(80)90213-8 6111141

[22] Tchouassi DP, Bastos AD, Sole CL, Diallo M, Lutomiah J, Mutisya J, et al Population genetics of two key mosquito vectors of Rift Valley fever virus reveals new insights into the changing disease outbreak patterns in Kenya. PLoS Negl Trop Dis. 2014;8(12):e3364 10.1371/journal.pntd.0003364 25474018 PMC4256213

[23] Turell MJ, Linthicum KJ, Patrican LA, Davies FG, Kairo A, Bailey CL. Vector competence of selected African mosquito (Diptera: Culicidae) species for Rift Valley fever virus. Journal of Medical Entomology. 2008;45(1):102–8. 18283949 10.1603/0022-2585(2008)45[102:vcosam]2.0.co;2

[24] Herve G. Enzootic activity of Rift Valley fever virus in Senegal. American Journal Tropical Medicine and Hygiene. 1997;56:265–72. 10.4269/ajtmh.1997.56.265 9129528

[25] Hoogstraal H, Meegan JM, Khalil GM, Adham FK. The Rift Valley fever epizootic in Egypt 1977–1978 2. Ecological and entomological studies. Transactions of the Royal Society of tropical Medicine and Hygiene. 1979;73(6):624–9. 44038 10.1016/0035-9203(79)90005-1

[26] Turell MJ, Kay BH. Susceptibility of selected strains of Australian mosquitoes (Diptera: Culicidae) to Rift Valley fever virus. Journal of Medical Entomology. 1998;35(2):132–5. 9538572 10.1093/jmedent/35.2.132

[27] Turell MJ, Presley SM, Gad AM, Cope SE, Dohm DJ, Morrill JC, et al Vector competence of Egyptian mosquitoes for Rift Valley fever virus. The American Journal of Tropical Medicine and Hygiene. 1996;54(2):136–9. 8619436 10.4269/ajtmh.1996.54.136

[28] Ochieng C, Lutomiah J, Makio A, Koka H, Chepkorir E, Yalwala S, et al Mosquito-borne arbovirus surveillance at selected sites in diverse ecological zones of Kenya; 2007–2012. Virology Journal. 2013;10(1):140. 23663381 10.1186/1743-422X-10-140 PMC3669043

[29] Owange NO, Ogara WO, Affognon H, Peter GB, Kasiiti J, Okuthe S, Onyango-Ouma W, Landmann T, Sang R & Mbabu M (2014) Occurrence of rift valley fever in cattle in Ijara district, Kenya. Preventive Veterinary Medicine 117: 121–128. 10.1016/j.prevetmed.2014.08.008 25217406

[30] Mosomtai G, Evander M, Sandström P, Ahlm C, Sang R, Hassan OA, Affognon H, Landmann T. Association of ecological factors with Rift Valley fever occurrence and mapping of risk zones in Kenya. Int J Infect Dis. 2016; 46: 49–55 10.1016/j.ijid.2016.03.013 26996461

[31] http://www.birdlife.org/news/tana-delta-kenya.

[32] Lwande OW, Lutomiah J, Obanda V, Gakuya F, Mutisya J, Mulwa F, et al Isolation of tick and mosquito-borne arboviruses from ticks sampled from livestock and wild animal hosts in Ijara District, Kenya. Vector-Borne and Zoonotic Diseases. 2013;13(9):637–42. 10.1089/vbz.2012.1190 23805790 PMC3777299

