# Supplementary material for: Distribution and abundance of key vectors of Rift Valley fever and other arboviruses in two ecologically distinct counties in Kenya
Source: PLoS Negl Trop Dis. 2017 Feb 17;11(2):e0005341. doi: 10.1371/journal.pntd.0005341 (PMC5333903; doi:10.1371/journal.pntd.0005341)
Supplement: S1 File — (DOCX) [file pntd.0005341.s001.docx]

**Primers used to characterize virus isolates**

| **Virus** | **Protein target** | **Primer sequence** | **Position** |
| --- | --- | --- | --- |
| Alphavirus | NSP4 | VIR 2052 F; (5′-TGG CGC TAT GAT GAA ATC TGG AAT GTT-3’) | 6971–6997 |
| " | " | VIR 2052R; (5′-TAC GAT GTT GTC GTC GCC GAT GAA-3′) | 7086–7109 |
| Flavivirus | NS5 | FU 1; (5′- TAC AAC ATG ATG GGA AAG AGA GAG AA-3′) | 9007-9032 |
| " | " | CFD2; (5′- GTG TCC CAG CCG GCG GTG TCA TCA GC-3′) | 9308-9283 |
| Bunya S | nucleocapsid protein | Bunya 1; (5'-GTCACAGTAGTGTACTCCAC-3') | 5-20 |
| " | " | Bunya 2; (5'-CTGACAGTAGTGTGCTCCAC-3') | 946-961 |
| RVFV | Glycoprotein M gene | RVF1; (5′-GAC TAC CAG TCA GCT CAT TAC C-3′) | 777-798 |
| " | " | RVF2; (5′-TGT GAA CAA TAG GCA TTG G-3′) | 1309-1327 |
| Sindbis | Non Structural Protein | SINV1; (5′-TTTAGCGGATCGGACAATTC-3′) | 5194-5213 |
| " | " | SINV2; (5′-GCGGTGACGAACTCAGTAG-3′) | 6482-6500 |
| Babanki | E1 envelope glycoprotein | Bab 3368 F; (5′- CAG CAG ATT GCG CGA CTG ACC-3′) | 3368-3388 |
| " | " | Bab 4203R; (5′- GCT CAC GAT ATG GTC AGC AGG-3′) | 4184-4203 |
| WNV | NS5 | FU 1; (5′- TAC AAC ATG ATG GGA AAG AGA GAG AA-3′) | 9007-9032 |
| " | " | CFD2; (5′- GTG TCC CAG CCG GCG GTG TCA TCA GC-3′) | 9308-9283 |
